# Phenotype Refinement Strengthens the Association of *AHR* and *CYP1A1* Genotype with Caffeine Consumption

**DOI:** 10.1371/journal.pone.0103448

**Published:** 2014-07-30

**Authors:** George McMahon, Amy E. Taylor, George Davey Smith, Marcus R. Munafò

**Affiliations:** 1 Medical Research Council Integrative Epidemiology Unit at the University of Bristol, Bristol, United Kingdom; 2 School of Social and Community Medicine, University of Bristol, Bristol, United Kingdom; 3 United Kingdom Centre for Tobacco and Alcohol Studies, University of Bristol, Bristol, United Kingdom; 4 School of Experimental Psychology, University of Bristol, Bristol, United Kingdom; University of Cincinnati, United States of America

## Abstract

Two genetic loci, one in the cytochrome P450 1A1 (*CYP1A1*) and 1A2 (*CYP1A2*) gene region (rs2472297) and one near the aryl-hydrocarbon receptor (*AHR*) gene (rs6968865), have been associated with habitual caffeine consumption. We sought to establish whether a more refined and comprehensive assessment of caffeine consumption would provide stronger evidence of association, and whether a combined allelic score comprising these two variants would further strengthen the association. We used data from between 4,460 and 7,520 women in the Avon Longitudinal Study of Parents and Children, a longitudinal birth cohort based in the United Kingdom. Self-report data on coffee, tea and cola consumption (including consumption of decaffeinated drinks) were available at multiple time points. Both genotypes were individually associated with total caffeine consumption, and with coffee and tea consumption. There was no association with cola consumption, possibly due to low levels of consumption in this sample. There was also no association with measures of decaffeinated drink consumption, indicating that the observed association is most likely mediated via caffeine. The association was strengthened when a combined allelic score was used, accounting for up to 1.28% of phenotypic variance. This was not associated with potential confounders of observational association. A combined allelic score accounts for sufficient phenotypic variance in caffeine consumption that this may be useful in Mendelian randomization studies. Future studies may therefore be able to use this combined allelic score to explore causal effects of habitual caffeine consumption on health outcomes.

## Introduction

Caffeine is one of the most widely-consumed psychoactive substances world-wide, and while coffee and tea consumption dominate, it is also present in some soft drinks [1]. There is also considerable inter-individual variability in preference for caffeine [2], in part due to genetic factors. Twin studies have consistently indicated substantial (∼50%) heritability of caffeine consumption (typically assessed as coffee consumption) [3]–[9]. Recently, a number of genome-wide association studies have identified variants robustly associated with caffeine consumption (again, typically assessed as coffee consumption) [10]–[12]. In particular, two loci, one in the cytochrome P450 1A1 (*CYP1A1*) and 1A2 (*CYP1A2*) gene region on chromosome 15 and one near the aryl-hydrocarbon receptor (*AHR*) gene on chromosome 7, have been found to be associated with habitual caffeine consumption across a number of studies [10]–[13]. Two single nucleotide polymorphisms, rs2472297 in between *CYP1A1* and *CYP1A2*, and rs6968865 51 kb upstream of *AHR*, provide the strongest signals, each with an effect equivalent to an increased consumption of ∼0.2 cups per day per risk (T) allele. The genes are biologically plausible candidates for caffeine consumption phenotypes as they both encode members of the same biochemical pathway. AHR is known to induce *CYP1A1* and *CYP1A2* by binding to the DNA in the region between these two genes [12], and low CYP1A2 activity has been associated with higher caffeine toxicity [14].

A limitation of studies to date is that they have typically used a single measure of caffeine consumption (e.g., coffee). One study [11] measured total caffeine consumption, but coffee contributed towards 80% of this, and data on other sources of caffeine were not reported separately. While coffee represents the major source of caffeine consumption in some countries, other sources of caffeine can be important. We have previously shown that phenotypic assessments which more accurately capture the exposure of interest can improve the precision of genetic association studies [15], particularly when the exposure (e.g., caffeine consumption) is strongly influenced by behaviour or behavioural choices (e.g., preference for coffee or tea). We therefore sought to establish whether using a more comprehensive phenotypic assessment of caffeine consumption, using measures of coffee, tea and cola consumption, would provide stronger evidence of association with rs2472297 and rs6968865. We were also interested in whether a combined allelic score comprising these two variants would further strengthen the association with caffeine consumption.

## Materials and Methods

### Study Sample

The Avon Longitudinal Study of Parents and Children (ALSPAC) sample is a longitudinal birth cohort that comprises 20,248 pregnancies. The mothers of 14,541 (71.8%) pregnancies were recruited antenatally during 1990–92 (Phase I). Post-natal recruitment to the ‘Focus@7’ clinical assessment at the age of ∼7 years recruited a further 456 children from 452 (2.2% of eligible) pregnancies (Phase II). Recruitment during ages 8–18 years (Phase III) added a further 257 children from 254 (1.2% of eligible) pregnancies, giving an overall total of 15,247 (75.3% of eligible) enrolled pregnancies; from these pregnancies there were 14,775 live-born children of which 14,701 were alive at one year of age. The phases of enrolment are described in more detail in the cohort profile paper [16]. The ALSPAC website contains details of all the data that are available through a fully searchable data dictionary: http://www.bristol.ac.uk/alspac/researchers/data-access/data-dictionary/. Ethics approval for the study was obtained from the ALSPAC Ethics and Law Committee and the Local Research Ethics Committees (Bristol and Weston Health Authority, Southmead Health Authority, Frenchay Health Authority).

### Measures of Caffeine Consumption

Data on coffee and tea consumption were collected via self-report during pregnancy at 8, 18 and 32 weeks gestation and 2, 47, 85, 97 and 145 months after delivery. Participants were asked to report “current daily coffee and tea drinking”, as number of drinks, separately for weekdays and weekends. Similar questions were asked for cola consumption in drinks per week. For cola consumption, questions were open format at 8, 18, and 32 weeks gestation, and 2 months after delivery, and closed format at later time points (“never or rarely”, “once in 2 weeks”, “1 to 3 times a week”, “4 to 7 times a week”, “once a day or more”). Closed format responses were recoded to 0, 0.5, 2, 5.5 and 7 drinks per week, and cola consumption values further recoded to reflect daily consumption. Outlying daily consumption values (>10 drinks for coffee, >15 drinks for tea and >21 drinks for cola) were coded as missing data. Similar questions were also asked for decaffeinated coffee, tea and cola consumption at the same time points, and coded in the same way. In order to obtain a measure of total daily caffeine consumption, number of cups of tea and coffee were summed with drinks per day of cola, weighted with respect to approximate caffeine content (coffee 75; tea 40; cola 34.5) [17], [18]. The distribution of total caffeine consumption, and coffee and tea consumption, is shown in Figures S1–S3.

### Genotyping

Genotypes at the *CYP1A1* (rs2472297) and *AHR* (rs6968865) loci were available from GWAS genotyping data. A total of 10,015 ALSPAC mothers were genotyped on the Illumina 660K quad chip at the Centre National de Genotypage, Paris, resulting in 557,124 directly genotyped SNPs before quality control. Genotypes were called with Illumina GenomeStudio and PLINK (v1.07) was used to carry out quality control steps.

Individuals were excluded from further analysis on the basis of having incorrect sex assignments; minimal or excessive heterozygosity, disproportionate levels of individual missingness (>5%); evidence of cryptic relatedness (>10% identical by descent) and being of non-European ancestry (as detected by a multidimensional scaling analysis seeded with HapMap 2 individuals). SNPs with a minor allele frequency of <1% and call rate of <95% were removed. Furthermore, only SNPs which passed an exact test of Hardy–Weinberg equilibrium (*P*>5×10^−6^) were considered for further use. Population stratification was assessed by means of multidimensional scaling of genome-wide identity by state (IBS) pairwise distances using the four (YOR, CEU, CHB, JPT) HapMap populations as a reference. Cryptic relatedness was assessed using estimates of the proportion of SNPs expected to be identical by descent given estimates of IBS. Subject with a relatedness of 0.1 or higher were excluded. Genotypes were imputed with Markov Chain Haplotyping software (MaCH 1.0.16) (45) using CEPH individuals from phase 2 of the HapMap project as a reference set (release 22). SNP rs2472297 was directly genotyped, had a MAF of 0.27, HWE *P*-value of 0.1 and 0.02% missingness before imputation. SNP rs6968865 was imputed with an imputation quality of 0.96, and MAF of 0.39. After imputation genotypes were available for 8,340 subjects. The frequencies of the T allele were 0.27 in rs2472297 and 0.61 in rs6968865.

### Statistical Analysis

Data on total caffeine consumption, and consumption of tea, coffee, cola and their decaffeinated counterparts, were analysed in a linear regression on number of T alleles in a univariate analysis of each SNP. Linear regression was carried out using the lm package in R (v. 2.14.0). Best-guess genotypes were used for analysis.

To obtain joint effects to take into account genotypes at both SNPs simultaneously, following Sulem and colleagues [12], the number of T alleles were summed across SNPs to derive a combined SNP score of the total number of T alleles per subject which was then used in a regression with phenotype data. For rs6968865 the T allele is the major allele, so that the SNP score contained one minor allele and one major (i.e., reference) allele. Weighting alleles using effect sizes obtained from Sulem and colleagues [12] (rs2472297 by 0.31, rs6968865 by 0.26) provided similar results and we present the results for the unweighted SNP score for simplicity.

We examined within-locus non-additivity by testing the significance of a second heterozygote term, and between-locus non-additivity by testing for a joint effect beyond the sum of the effects of both SNPs individually. Our results indicated that these SNPs act additively, and their effects are independent (although we cannot rule out more complicated interactions between these SNPs in the presence of other factors).

Data used for this submission will be made available on request to the ALSPAC executive committee (alspac-exec@bristol.ac.uk). The ALSPAC data management plan (available here: http://www.bristol.ac.uk/alspac/researchers/data-access/) describes in detail the policy regarding data sharing, which is through a system of managed open access.

## Results

### Characteristics of Participants

**Figure 1:**
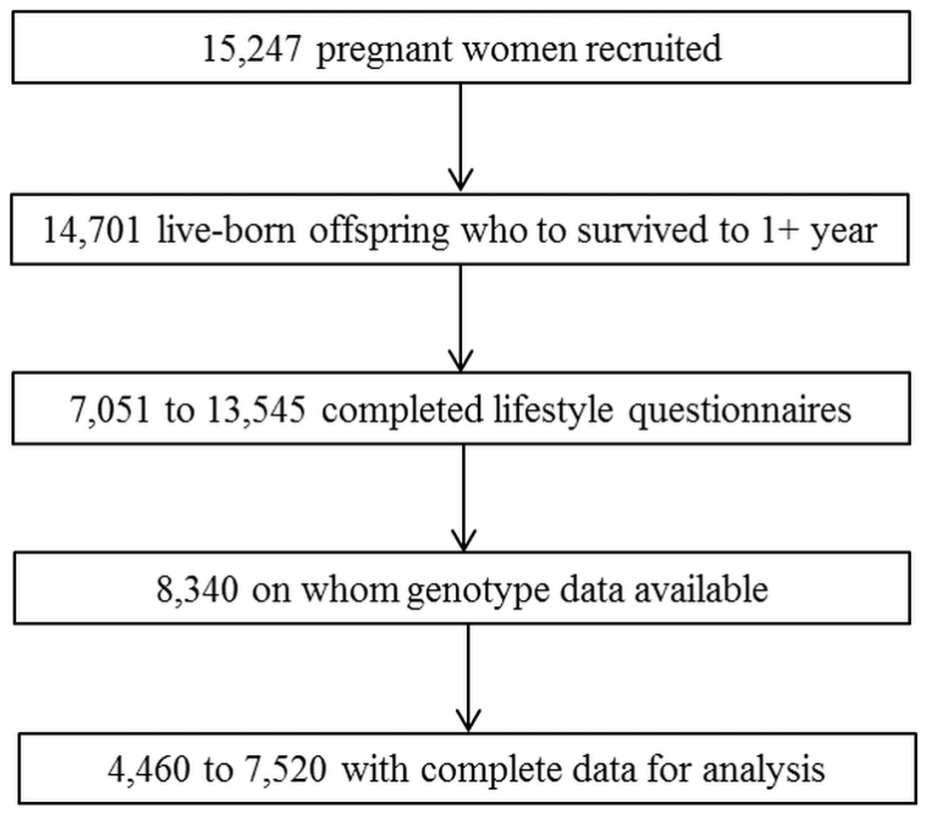
Study Participant Flow Diagram. Due to study attrition, data obtained when the cohort first started have a higher number of responses than variables collected later. Thus the number of participants on whom data are available is given as a range.

The total sample available for analysis comprised between 4,460 and 7,520 women (see Figure 1 for a summary of how this sample was arrived at). Levels of missingness were low unless questions on caffeine consumption were not included in one or more versions of the questionnaire at that time point. More information on ALSPAC mothers' response rates has been published previously [16].

Consumption of coffee tended to increase roughly linearly across time points (means 1.18 to 2.30 drinks per day). Consumption of tea (means 2.73 to 3.18 drinks per day) and cola (means 0.60 to 2.31 drinks per week) varied across time points, but with no clear pattern of change. As a result, total daily caffeine consumption tended to increase across time points (means 206.8 mg to 306.1 mg). These data are shown in Tables 1–4. In general, cola consumption was considerably less than tea and coffee consumption, reflecting approximately 4% to 11% of total caffeine consumption in drinks per day.

**Table 1:** Association of *CYP1A1* rs2472297, *AHR* rs6968865 and combined SNP score with total caffeine consumption (mg).

Time	N	Mean	SD	Min	Max	*CYP1A1* rs2472297			*AHR* rs6968865			Combined Score		
						Beta	SE	P-Value	Beta	SE	P-Value	Beta	SE	P-Value
8 wk	6785	206.8	142.4	0	1201	8.7	2.7	1.59×10^−03^	4.0	2.5	1.15×10^−01^	5.9	1.8	1.15×10^−03^
18 wk	7356	215.3	142.4	0	1161	12.4	2.6	2.29×10^06^	7.9	2.4	1.08×10^−03^	9.7	1.8	2.94×10^−08^
32 wk	6898	216.0	141.5	0	1150	14.6	2.7	5.85×10^−08^	5.6	2.5	2.36×10^−02^	9.5	1.8	1.31×10^−07^
2 mo	4659	233.5	154.8	0	1350	11.8	3.6	9.54×10^−04^	7.7	3.3	1.90×10^−02^	9.3	2.4	9.74×10^−05^
47 mo	5894	302.5	149.6	0	1150	16.8	3.1	4.47×10^−08^	11.0	2.8	1.09×10^−04^	13.2	2.0	1.04×10^−10^
85 mo	5199	306.1	150.5	0	1152	16.0	3.3	1.05×10^−06^	13.7	3.0	6.34×10^−06^	14.2	2.2	7.38×10^−11^
97 mo	4958	299.3	147.1	0	1160	15.8	3.3	1.49×10^−06^	10.9	3.0	3.04×10^−04^	12.7	2.2	5.80×10^−09^
145 mo	4460	278.3	144.9	0	1185	21.4	3.4	3.33×10^−10^	14.6	3.1	3.34×10^−06^	17.1	2.3	3.74×10^−14^

Caffeine consumption (mg) calculated as the sum of number of cups of tea and coffee, and drinks of cola, per day, weighted by approximate caffeine content. Time reflects data collected during pregnancy at 8, 18 and 32 weeks gestation and 2, 47, 85, 97 and 145 months after delivery. Beta reflects the number of drinks per day per T allele. Combined Score reflects the number of T alleles summed across SNPs rs2472297 and rs6968865.

**Table 2:** Association of *CYP1A1* rs2472297, *AHR* rs6968865 and combined SNP score with coffee consumption.

Time	N	Mean	SD	Min	Max	*CYP1A1* rs2472297			*AHR* rs6968865			Combined Score		
						Beta	SE	P-Value	Beta	SE	P-Value	Beta	SE	P-Value
8 wk	7102	1.18	1.66	0	10	0.069	0.031	2.62×10^−02^	0.031	0.029	2.79×10^−01^	0.047	0.021	2.34×10^−02^
18 wk	7520	1.25	1.68	0	10	0.082	0.031	7.47×10^−03^	0.078	0.028	5.39×10^−03^	0.078	0.020	1.36×10^−04^
32 wk	7076	1.24	1.66	0	10	0.110	0.031	4.35×10^−04^	0.037	0.029	1.99×10^−01^	0.068	0.021	1.02×10^−03^
2 mo	4774	1.47	1.92	0	10	0.105	0.044	1.67×10^−02^	0.107	0.040	8.31×10^−03^	0.102	0.029	4.59×10^−04^
47 mo	5953	2.26	2.14	0	10	0.134	0.044	2.22×10^−03^	0.111	0.040	6.09×10^−03^	0.118	0.029	5.46×10^−05^
85 mo	5279	2.30	2.16	0	10	0.081	0.047	8.42×10^−02^	0.096	0.043	2.74×10^−02^	0.086	0.031	6.10×10^−03^
97 mo	5046	2.22	2.11	0	10	0.096	0.047	4.06×10^−02^	0.099	0.043	2.07×10^−02^	0.095	0.031	2.35×10^−03^
145 mo	4532	2.05	1.97	0	10	0.113	0.046	1.34×10^−02^	0.132	0.042	1.76×10^−03^	0.120	0.031	9.01×10^−05^

Coffee consumption calculated as the number of cups of coffee per day. Time reflects data collected during pregnancy at 8, 18 and 32 weeks gestation and 2, 47, 85, 97 and 145 months after delivery. Beta reflects the number of drinks per day per T allele. Combined Score reflects the number of T alleles summed across SNPs rs2472297 and rs6968865.

**Table 3:** Association *CYP1A1* rs2472297, *AHR* rs6968865 and combined SNP score with tea consumption.

Time	N	Mean	SD	Min	Max	*CYP1A1* rs2472297			*AHR* rs6968865			Combined Score		
						Beta	SE	P-Value	Beta	SE	P-Value	Beta	SE	P-Value
8 wk	7120	2.73	2.30	0	15	0.103	0.043	1.74×10^−02^	0.057	0.040	1.48×10^−01^	0.076	0.029	8.31×10^−03^
18 wk	7515	2.78	2.28	0	15	0.133	0.042	1.40×10^−03^	0.052	0.038	1.76×10^−01^	0.087	0.028	1.80×10^−03^
32 wk	7056	2.95	2.27	0	15	0.133	0.043	1.83×10^−03^	0.081	0.039	3.99×10^−02^	0.102	0.028	3.45×10^−04^
2 mo	5340	3.05	2.42	0	15	0.128	0.052	1.42×10^−02^	0.040	0.048	4.11×10^−01^	0.077	0.035	2.58×10^−02^
47 mo	5957	3.17	2.48	0	15	0.170	0.051	7.48×10^−04^	0.084	0.047	7.22×10^−02^	0.120	0.034	3.82×10^−04^
85 mo	5303	3.18	2.52	0	15	0.239	0.054	1.01×10^−05^	0.157	0.050	1.79×10^−03^	0.187	0.036	2.08×10^−07^
971mo	5051	3.18	2.47	0	15	0.224	0.055	4.21×10^−05^	0.083	0.050	1.00×10^−01^	0.143	0.036	9.01×10^−05^
145 mo	4632	2.98	2.38	0	15	0.317	0.055	6.80×10^−09^	0.127	0.051	1.24×10^−02^	0.209	0.037	1.23×10^−08^

Tea consumption calculated as the number of cups of tea per day. Time reflects data collected during pregnancy at 8, 18 and 32 weeks gestation and 2, 47, 85, 97 and 145 months after delivery. Beta reflects the number of drinks per day per T allele. Combined Score reflects the number of T alleles summed across SNPs rs2472297 and rs6968865.

**Table 4:** Association of *CYP1A1* rs2472297, *AHR* rs6968865 and combined SNP score with cola consumption.

Time	N	Mean	SD	Min	Max	*CYP1A1* rs2472297			*AHR* rs6968865			Combined Score		
						Beta	SE	P-Value	Beta	SE	P-Value	Beta	SE	P-Value
8 wk	6876	2.31	4.20	0	21	0.116	0.080	1.50×10^−01^	−0.113	0.074	1.23×10^−01^	−0.009	0.054	8.68×10^−01^
18 wk	7412	2.12	4.13	0	21	0.078	0.076	3.07×10^−01^	−0.019	0.070	7.89×10^−01^	0.025	0.051	6.25×10^−01^
32 wk	6976	1.31	2.50	0	21	0.014	0.047	7.73×10^−01^	−0.027	0.043	5.28×10^−01^	−0.008	0.032	7.91×10^−01^
2 mo	5243	0.60	1.66	0	21	−0.021	0.036	5.64×10^−01^	−0.071	0.033	3.43×10^−02^	−0.046	0.024	5.51×10^−02^
47 mo	6023	1.47	2.10	0	7	0.004	0.043	9.26×10^−01^	−0.064	0.039	1.07×10^−01^	−0.032	0.029	2.69×10^−01^
85 mo	5315	1.36	2.03	0	7	−0.019	0.044	6.61×10^−01^	0.010	0.041	7.96×10^−01^	−0.003	0.029	9.15×10^−01^
97 mo	5096	1.33	2.00	0	7	0.035	0.044	4.34×10^−01^	0.032	0.041	4.27×10^−01^	0.032	0.029	2.73×10^−01^
145 mo	4685	0.97	1.72	0	7	0.029	0.039	4.61×10^−01^	0.018	0.036	6.25×10^−01^	0.022	0.026	3.96×10^−01^

Cola consumption calculated as the number of drinks of cola per week. Time reflects data collected during pregnancy at 8, 18 and 32 weeks gestation and 2, 47, 85, 97 and 145 months after delivery. Beta reflects the number of drinks per week per T allele. Combined Score reflects the number of T alleles summed across SNPs rs2472297 and rs6968865.

### Caffeine Consumption

Across all time points, total caffeine consumption was associated with both *CYP1A1* (βs  = 8.7 to 21.4, *P*s  = 1.59×10^−3^ to 3.33×10^−10^) and *AHR* (βs  = 4.0 to 14.6, *P*s  = 1.15×10^−1^ to 3.34×10^−6^) genotypes (Table 1). Similarly, total caffeine consumption was also associated with the combined SNP score, and the statistical evidence for this association considerably stronger (βs  = 5.9 to 17.1, *P*s  = 1.15×10^−3^ to 3.74×10^−14^).

In general, the proportion of phenotypic variance explained across all time points was small, as would be expected for the association of common variants with complex behavioural phenotypes. For *CYP1A1*, the proportion of phenotypic variance explained ranged from 0.15% to 0.88%, while for *AHR* it ranged from 0.04% to 0.48%. However, the combined SNP score accounted for a somewhat higher proportion of phenotypic variance on average, ranging from 0.16% to 1.28%.

Estimates of the proportion of phenotypic variance obtained using GCTA [19] for the two SNPs in the 2-SNP score were broadly similar to those obtained using linear regression (0.10% to 1.10% vs 0.16% to 1.28%). GCTA analysis for the remaining directly-genotyped SNPs available accounted for additional phenotypic variance, although these estimates may be unreliable due to relatively small sample size (see Table S1).

Stratified analyses further indicated that these associations were present for consumption of coffee (combined SNP score: βs  = 0.047 to 0.120, *P*s  = 2.34×10^−2^ to 5.46×10^−5^) and tea (combined SNP score: βs  = 0.076 to 0.209, *P*s  = 2.58×10^−2^ to 1.23×10^−8^), but not cola (combined SNP score: βs  = −0.046 to 0.032, *P*s  = 9.15×10^−1^ to 5.51×10^−2^) (Tables 2–4). Interestingly, associations for tea consumption were generally stronger than for coffee consumption. Removing participants who reported zero consumption of coffee, tea and/or cola did not alter these results substantially.

There was no evidence that either *AHR* or *CYP1A1* genotypes, or the combined SNP score, was associated with consumption of decaffeinated coffee, tea or cola (see Tables S2–S4), indicating that the associations observed are specific to caffeinated drinks. Again, removing participants who reported zero consumption of coffee, tea and/or cola did not alter these results substantially. We also did not observe any association with measures of aversion to coffee, tea or cola taken during pregnancy (data available on request).

### Potential Confounders

Next we assessed the association of the combined SNP score with potential confounders (year of birth, educational attainment, measures of socioeconomic position, alcohol use, tobacco use). These indicated no evidence of association (Table 5), suggesting that the combined SNP score may be a useful instrumental variable in Mendelian randomization analyses [20], [21]. This is in contrast with the association of total caffeine consumption with the same potential confounders, which shows very strong evidence of association at multiple time points (Table 6). A full description of these variables is provided in the ALSPAC cohort profile [16].

**Table 5:** Association of combined SNP score with potential confounders.

	N	*CYP1A1* rs2472297			*AHR* rs6968865			Combined		
		Beta	SE	P-Value	Beta	SE	P-Value	Beta	SE	P-Value
Year of Birth	7882	−0.003	0.085	0.976	−0.048	0.078	0.537	−0.027	0.057	0.639
Housing Tenure	7300	0.016	0.013	0.206	−0.005	0.012	0.653	0.004	0.009	0.603
Crowding Index	7434	0.009	0.007	0.195	−0.003	0.007	0.658	0.003	0.005	0.588
Educational Level	6919	−0.009	0.023	0.683	−0.011	0.021	0.604	−0.010	0.015	0.515
Alcohol Consumption	7507	0.043	0.068	0.531	−0.029	0.063	0.643	0.004	0.046	0.934
Tobacco Consumption	7552	0.130	0.095	0.172	0.024	0.087	0.783	0.071	0.063	0.265

Housing tenure was coded as: bought/mortgaged/owned with no mortgage to pay, rented from private landlord, rented from council/housing association. Crowding index was coded as number of people living in household divided by the number of rooms. Highest educational level was coded as the equivalent of: none, vocational, school to age 16, school to age 18, degree or higher. Alcohol consumption was measured in drinks per week. Tobacco consumption was measured in times per day. Linearity was imposed on the categorical variables (housing tenure, educational level). Measures of alcohol and tobacco consumption shown were taken at 18 weeks gestation, but results were similar at the other time points.

**Table 6:** Association of total caffeine consumption (mg) with potential confounders.

	N	Time								
		18 week			47 month			145 month		
		Beta	SE	P-Value	Beta	SE	P-Value	Beta	SE	P-Value
Year of Birth	7346–4320	0.780	0.352	2.70×10^−02^	−2.63	0.426	7.27×10^−10^	−0.538	0.502	2.83×10^−01^
Housing Tenure	6951–4134	26.60	2.497	2.71×10^−26^	19.33	3.207	1.78×10^−09^	16.51	4.090	5.53×10^−05^
Crowding Index	7063–4194	29.59	4.360	1.26×10^−11^	20.19	5.717	4.15×10^−04^	14.45	6.737	3.20×10^−02^
Educational Level	6637–4117	−14.97	1.430	1.83×10^−25^	−3.026	1.725	8.00×10^−02^	−6.54	1.988	1.00×10^−03^
Alcohol Consumption	7334–4244	3.958	0.461	1.18×10^−17^	2.673	0.571	2.94×10^−06^	1.180	0.681	8.30×10^−02^
Tobacco Consumption	7331–4267	7.908	0.315	4.13×10^−133^	6.870	0.422	2.48×10^−58^	6.317	0.542	6.74×10^−31^

Housing tenure was coded as: bought/mortgaged/owned with no mortgage to pay, rented from private landlord, rented from council/housing association. Crowding index was coded as number of people living in household divided by the number of rooms. Highest educational level was coded as the equivalent of: none, vocational, school to age 16, school to age 18, degree or higher. Alcohol consumption was measured in drinks per week. Tobacco consumption was measured in times per day. Linearity was imposed on the categorical variables (housing tenure, educational level). Measures of alcohol and tobacco consumption shown were taken at 18 weeks gestation, but results were similar at the other time points.

## Discussion

Our results confirm that two SNPs in *AHR* and *CYP1A1* are associated with caffeine consumption, and extend previous findings in two important ways. First, our results are the first to show association in a sample where caffeine consumption via caffeinated beverages other than coffee is common. Moreover, we show that a combined caffeine consumption phenotype derived from measures of consumption of three caffeinated beverages (coffee, tea and cola) provides a stronger signal than any one of these measures separately. Second, our results also confirm that these results are due to caffeine consumption, rather than some other common characteristic of caffeinated beverages. By using measures of consumption of decaffeinated drinks as negative controls we show no evidence of association with either *AHR* or *CYP1A1*. While our results hold for both SNPs individually, our strongest results are obtained when both SNPs are combined to create a 2-SNP genetic risk score.

Observationally, caffeine (or, more commonly, coffee) consumption has been shown to be associated with a number of health outcomes [22]. Evidence from longitudinal studies suggests that long-term coffee consumption may in fact be protective against cardiovascular disease [22], [23] and lower the risk of all-cause mortality [24]. Coffee consumption also shows an inverse association with diabetes, although this may be due to antioxidant compounds within coffee rather than caffeine itself [23]. Observational studies suggest that coffee consumption may have further beneficial health effects, including reducing risk of several cancers, such as endometrial, liver and prostate cancer [25]–[27] and protecting against depression, attention deficit hyperactivity disorder and Alzheimer disease [28]–[30]. Conversely, it is recommended that caffeine consumption is restricted during pregnancy due to its association with adverse pregnancy outcomes such as intrauterine growth retardation and miscarriage [31], [32]. Observational studies also suggest that caffeine consumption may be detrimental to bone health, leading to increased fracture risk [33]. However, these studies all suffer from the usual problems of residual confounding and reverse causality which limit the causal inferences that can be drawn from observational data.

Mendelian randomization (MR) offers one approach to better understanding the causal nature of the observed associations between caffeine consumption and health outcomes. Genetics variants are randomly assorted during gamete formation and conception, and therefore should be unrelated to other lifestyle factors associated with coffee consumption which may confound observational associations [34]. Health outcomes cannot affect the genes that an individual has, so we know that associations from MR analyses are not due to reverse causality [34]. This may be particularly important in observational studies of the effects of caffeine as individuals may alter levels of caffeine consumption in response to ill health. In addition, caffeine consumption is difficult to measure accurately as it is usually obtained from food frequency questionnaires [35], so observational estimates may be biased by random or non-random measurement error. In contrast, MR can provide accurate estimates of the magnitude of lifelong exposure to a risk factor [36].

Critically, we have shown that the two SNPs in *AHR* and *CYP1A1*, and our 2-SNP genetic risk score, are not associated with a range of potential confounders that may give rise to spurious associations in studies of health-outcomes putatively related to caffeine consumption. This, together with the clear evidence of association with caffeine consumption, indicates that the 2-SNP genetic risk score could be used as an instrumental variable in MR analyses. The greater variance explained by the combined score would increase statistical power and reduce the sample size required to detect associations with health outcomes, compared to using either SNP individually. The risk score explains up to 1.3% of the variance in caffeine consumption, which although small in absolute terms is relatively large by the standards of common genetic variants. This is comparable to the variance explained in body mass index (BMI) by variants in the *FTO* gene, and in cigarette consumption by variants in the *CHRNA5-A3-B4* gene cluster [15], [37], which have been used in MR studies of the causal effects of BMI and smoking on health outcomes [38]–[41]. The 2-SNP score for caffeine consumption may therefore be a suitable instrument to explore the causal effects of caffeine consumption on a range of health outcomes.

There are some limitations to this study that should be considered when interpreting our results. First, caffeine consumption was measured using a food frequency questionnaire, and these may have modest reliability and validity [35]. We were also only able to capture tea, coffee and cola drinks as sources of dietary caffeine, and not other sources (e.g., chocolate). However, tea, coffee and soft drinks (including cola) together account for ∼90% of caffeine consumption in similar populations, and the levels of consumption we observed are similar to those observed in other studies [32]. While more detailed assessments of caffeine consumption are possible, these are difficult to obtain on the scale necessary for genetic association studies. Future studies could obtain more detailed phenotypic information on selected, genetically-informative individuals [42]. Second, levels of cola consumption were low in this sample, so that this, together with the relatively low levels of caffeine in cola drinks, may account for the lack of association observed. It is also possible that participants were responding to questions about “cola” consumption at least in part as questions about *all* soda consumption. To better understand whether this lack of association is genuine will require the study of populations where levels of cola consumption are higher. Third, our sample was restricted to women only. Rates of caffeine consumption may differ between men and women, although there are no clear reasons to expect that the pattern of results we observed would differ in males. While patterns of consumption during pregnancy may not be typical, our data extend to ∼12 years post-pregnancy. It is likely that the women in our sample reverted to pre-pregnancy patterns of caffeine consumption over time. Fourth, we only included 2 SNPs in our analysis. These were chosen on the basis of being those for which there is the clearest evidence from recent GWAS of caffeine consumption. Future studies may extend our 2-SNP score by including further variants. Fifth, although we are optimistic that these genotypes, and the 2-SNP score, can be used as instrumental variables in MR analyses, potential pleiotropic effects will need to be considered. Metabolic enzyme genotypes typically relate to several metabolic differences with may give rise to associations with health outcomes. In principle, this can be tested by examining the association of genotype with health outcome separately in those who do and do not consume caffeinated drinks [43] – the genotype should not be associated with the outcome in the latter group if the association is mediated via caffeine consumption (although this can give rise to collider bias [44]). Finally, participants of non-European ancestry were excluded during preparation of GWAS data, given that differences in ancestry can bias genetic association studies. Therefore, genotypes were only available for participants of European ancestry. However, >95% of ALSPAC participants are of European ancestry, so we think it unlikely that this influenced our results.

In conclusion, our data confirm the association of *AHR* and *CYP1A1* genotypes with caffeine consumption, and extend previous work by showing that this association holds for tea consumption as well as coffee consumption. Moreover, no association is observed for decaffeinated tea or coffee consumption. This strengthens the argument that the association is mediated via caffeine consumption, although it remains possible that other compounds present in both tea and coffee mediate this association. Future work, perhaps selecting participants on the basis of *AHR* and *CYP1A1* genotype, could explore this possibility through the administration of caffeine in a laboratory setting. Finally, the relatively large proportion of variance in caffeine consumption accounted for by the combined SNP score, and the lack of association of this with potential confounders, means that it could be used in Mendelian randomization studies to explore the causal effects of habitual caffeine consumption on health-related outcomes.

## Supporting Information

Figure S1 **Distribution of total caffeine consumption (mg).**
(TIF)

Figure S2 **Distribution of total coffee consumption (cups per day).**
(TIF)

Figure S3 **Distribution of total tea consumption (cups per day).**
(TIF)

Table S1 **Variance in total caffeine consumption explained using linear regression and GCTA.**
(DOCX)

Table S2 **Association of** ***CYP1A1*** **rs2472297,** ***AHR*** **rs6968865 and combined genetic score with decaffeinated coffee consumption.**
(DOCX)

Table S3 **Association of** ***CYP1A1*** **rs2472297,** ***AHR*** **rs6968865 and combined genetic score with decaffeinated tea consumption.**
(DOCX)

Table S4 **Association of** ***CYP1A1*** **rs2472297,** ***AHR*** **rs6968865 and combined genetic score with decaffeinated cola consumption.**
(DOCX)
