# Supplementary figures and images for: Phenotype Refinement Strengthens the Association of AHR and CYP1A1 Genotype with Caffeine Consumption
Source: PLoS One. 2014 Jul 30;9(7):e103448. doi: 10.1371/journal.pone.0103448 (PMC4116211; doi:10.1371/journal.pone.0103448)

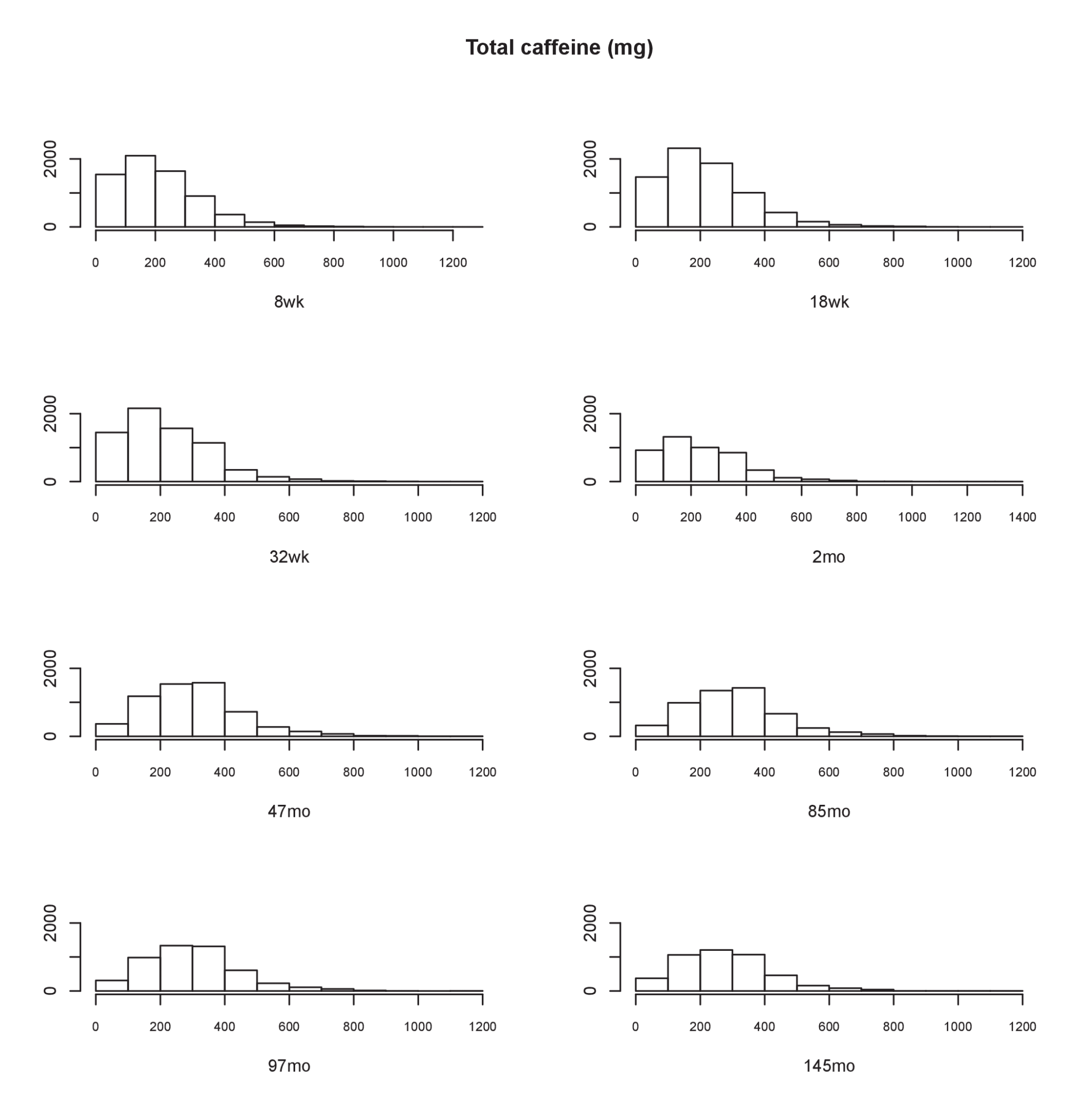

Supplement: Figure S1 — Distribution of total caffeine consumption (mg). (TIF) [file pone.0103448.s001.tif]

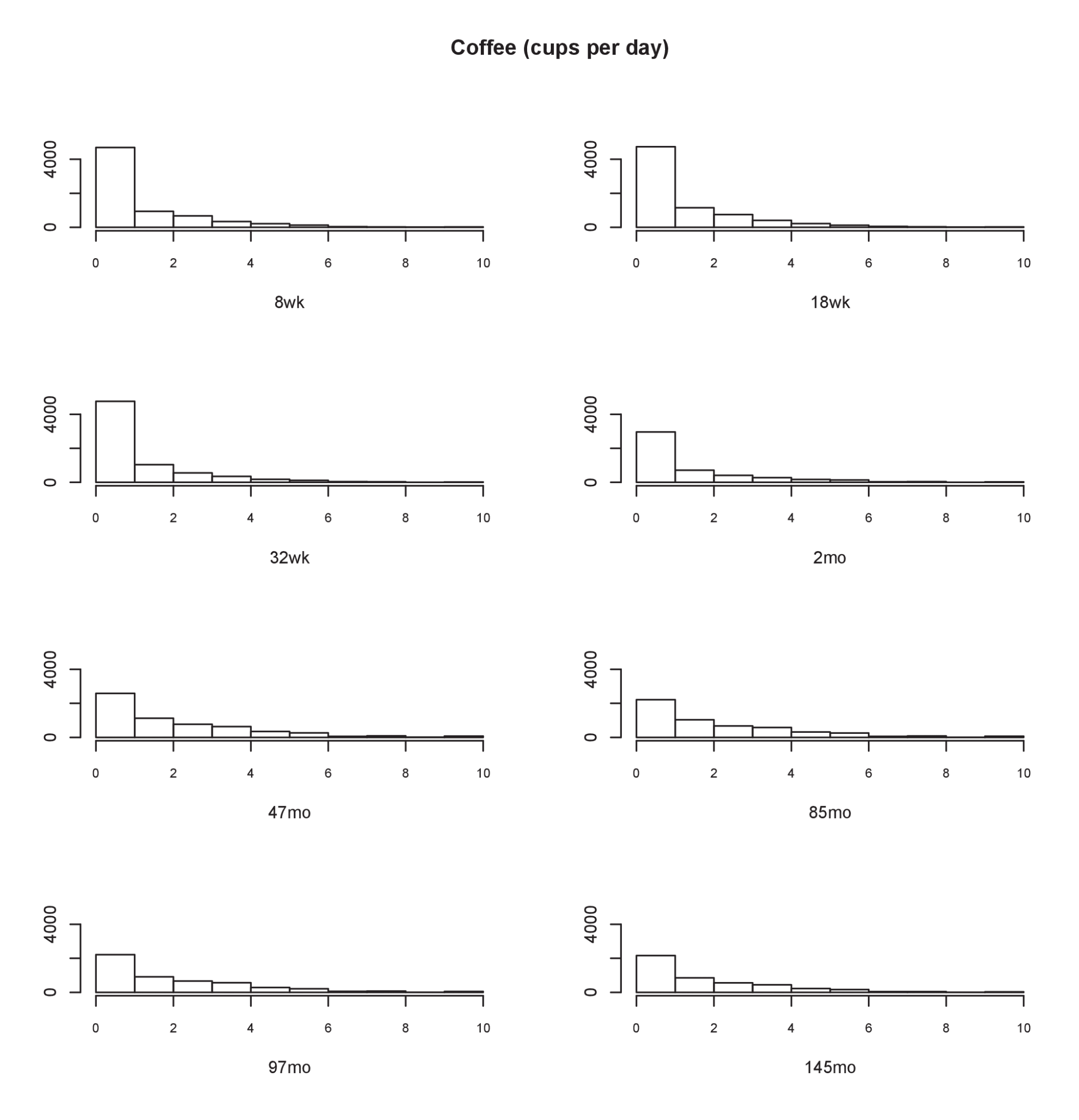

Supplement: Figure S2 — Distribution of total coffee consumption (cups per day). (TIF) [file pone.0103448.s002.tif]

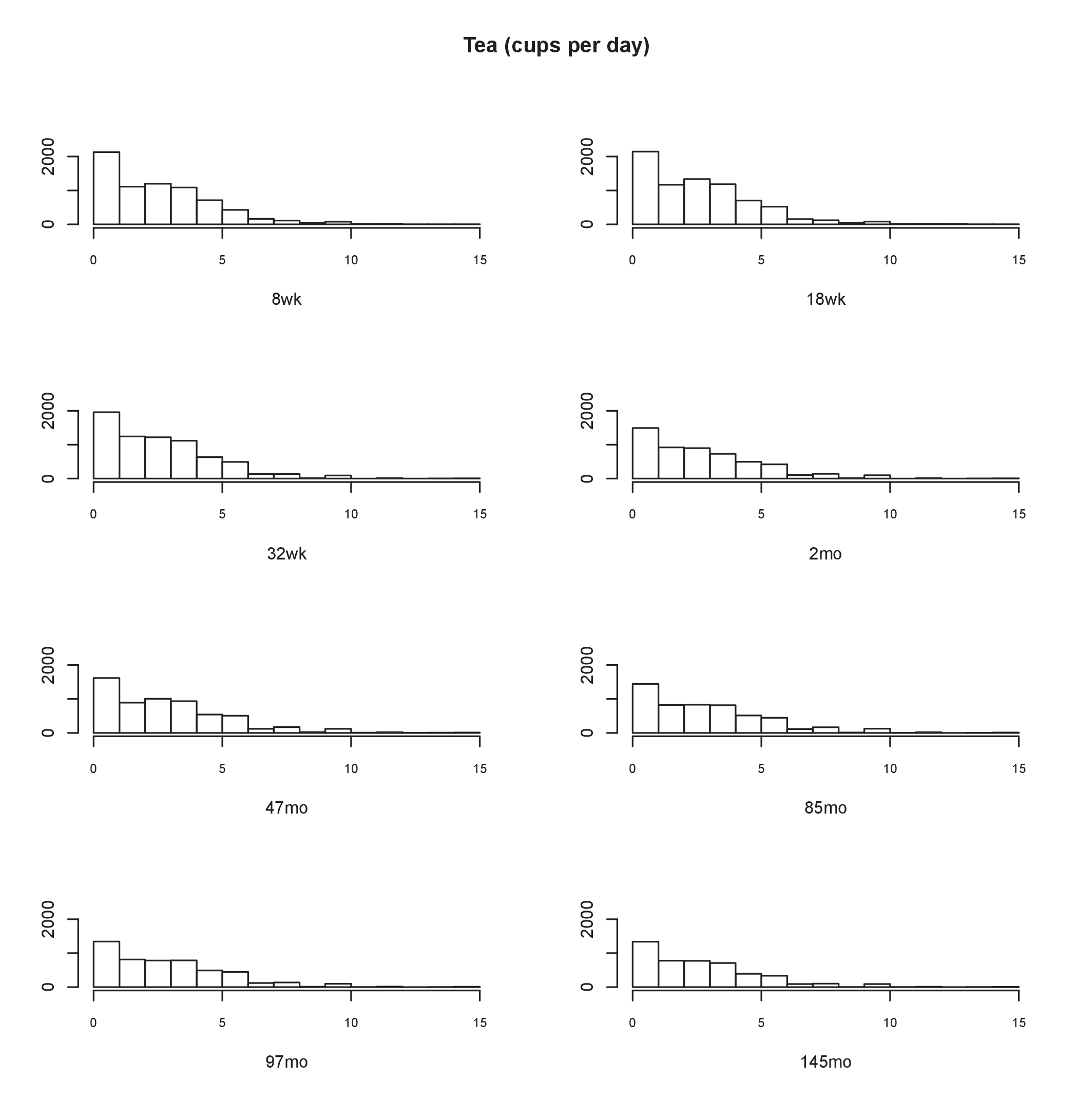

Supplement: Figure S3 — Distribution of total tea consumption (cups per day). (TIF) [file pone.0103448.s003.tif]
