# Supplementary material for: Phenotype Refinement Strengthens the Association of AHR and CYP1A1 Genotype with Caffeine Consumption
Source: PLoS One. 2014 Jul 30;9(7):e103448. doi: 10.1371/journal.pone.0103448 (PMC4116211; doi:10.1371/journal.pone.0103448)
Supplement: Table S1 — Variance in total caffeine consumption explained using linear regression and GCTA. (DOCX) [file pone.0103448.s004.docx]

Table S1. Variance in total caffeine consumption explained using linear regression and GCTA.

| **Timepoint** | **N** | **2-SNP Score r^2^**  **(SEs)** | **P-Value** | **N** | **V(G_2 SNPs_)/Vp**  **(SE)** | **P-Value** | **V(G_all other SNPs_)/Vp**  **(SE)** | **P-Value** |
| --- | --- | --- | --- | --- | --- | --- | --- | --- |
| 8wk | 6785 | 0.0016  (0.0010) | 1.0 × 10^-03^ | 6011 | 0.0010  (0.0013) | 4.0 × 10^-02^ | 0.070  (0.057) | 1.0 × 10^-01^ |
| 18wk | 7356 | 0.0042  (0.0015) | 3.0 × 10^-08^ | 6507 | 0.0031  (0.0034) | 4.0 × 10^-05^ | 0.051  (0.052) | 2.0 × 10^-01^ |
| 32wk | 6898 | 0.0040  (0.0015) | 1.0 × 10^-07^ | 6131 | 0.0035  (0.0038) | 2.0 × 10^-05^ | 0.147  (0.056) | 3.0 × 10^-03^ |
| 2mo | 4659 | 0.0033  (0.0017) | 1.0 × 10^-04^ | 4154 | 0.0020  (0.0025) | 1.0 × 10^-02^ | 0.062  (0.083) | 2.0 × 10^-01^ |
| 47mo | 5894 | 0.0071  (0.0022) | 1.0 × 10^-10^ | 5264 | 0.0072  (0.0075) | 5.0 × 10^-09^ | 0.232  (0.066) | 1.0 × 10^-04^ |
| 85mo | 5199 | 0.0081  (0.0025) | 7.0 × 10^-11^ | 4670 | 0.0076  (0.0080) | 1.0 × 10^-08^ | 0.261  (0.074) | 1.0 × 10^-04^ |
| 97mo | 4958 | 0.0068  (0.0023) | 6.0 × 10^-09^ | 4454 | 0.0062  (0.0066) | 6.0 × 10^-07^ | 0.055  (0.077) | 2.0 × 10^-01^ |
| 145mo | 4460 | 0.0128  (0.0033) | 4.0 × 10^-14^ | 4012 | 0.0110  (0.0114) | 1.0 × 10^-10^ | 0.087  (0.085) | 1.0 × 10^-01^ |

Variance in total caffeine consumption is shown, as explained by the 2-SNP score in linear regression (2-SNP Score r^2^), by the two SNPs using GCTA (V(G_2 SNPs_)/Vp), and by all remaining directly genotyped SNPs using GCTA (V(G_all other SNPs_)/Vp). GCTA (version 1.04) was used to obtain estimates of the variance explained by all remaining SNPs (after removal of SNPs rs2472297 and rs6968865). Estimates of the variance explained by the remaining SNPs included in the directly genotyped dataset were obtained by partitioning the variance explained by the 2 SNPs or all remaining SNPs using a two component mixed model $y= \mu+ \beta_{1}z_{i 2 SNP}+ \beta_{2}z_{i all other SNPs + \varepsilon_{i}}$ in GCTA. In all analyses, one of a pair of subjects with a relatedness of > 0.025 was excluded and the effect of including principal components generated in EIGENSTRAT (version 4.2) [[1](#_ENREF_1)] was examined.

Reference

1. Price, A.L., et al., *Principal components analysis corrects for stratification in genome-wide association studies.* Nat Genet, 2006. **38**(8): p. 904-9.
