# Supplementary material for: Phenotype Refinement Strengthens the Association of AHR and CYP1A1 Genotype with Caffeine Consumption
Source: PLoS One. 2014 Jul 30;9(7):e103448. doi: 10.1371/journal.pone.0103448 (PMC4116211; doi:10.1371/journal.pone.0103448)
Supplement: Table S3 — Association of CYP1A1 rs2472297, AHR rs6968865 and combined genetic score with decaffeinated tea consumption. (DOCX) [file pone.0103448.s006.docx]

Table S3. Association of *CYP1A1* rs2472297, *AHR* rs6968865 and combined genetic score with decaffeinated tea consumption.

| **Time** | **N** | **Mean** | **SD** | **Min** | **Max** | ***CYP1A1* rs2472297** | | | ***AHR* rs6968865** | | | **Combined Score** | | |
| --- | --- | --- | --- | --- | --- | --- | --- | --- | --- | --- | --- | --- | --- | --- |
|  |  |  |  |  |  | **Beta** | **SE** | **P-Value** | **Beta** | **SE** | **P-Value** | **Beta** | **SE** | **P-Value** |
| 8wk | 6990 | 0.09 | 0.57 | 0 | 11 | -0.009 | 0.011 | 0.38 | -0.009 | 0.010 | 0.37 | -0.009 | 0.007 | 0.22 |
| 18wk | 7532 | 0.09 | 0.59 | 0 | 11 | -0.005 | 0.011 | 0.65 | -0.001 | 0.010 | 0.91 | -0.003 | 0.007 | 0.70 |
| 32wk | 6838 | 0.17 | 0.82 | 0 | 12 | -0.015 | 0.016 | 0.33 | 0.006 | 0.014 | 0.67 | -0.004 | 0.010 | 0.73 |
| 2mo | 4877 | 0.19 | 0.90 | 0 | 12 | -0.035 | 0.020 | 0.08 | 0.057 | 0.019 | 0.002 | 0.014 | 0.013 | 0.30 |
| 47mo | 5979 | 0.15 | 0.81 | 0 | 15 | -0.007 | 0.016 | 0.68 | 0.019 | 0.015 | 0.21 | 0.007 | 0.011 | 0.54 |
| 85mo | 4737 | 0.14 | 0.80 | 0 | 12 | -0.006 | 0.018 | 0.76 | 0.026 | 0.017 | 0.12 | 0.011 | 0.012 | 0.37 |
| 97mo | 4690 | 0.15 | 0.81 | 0 | 11 | 0.005 | 0.018 | 0.80 | 0.010 | 0.017 | 0.55 | 0.007 | 0.012 | 0.55 |
| 145mo | 4483 | 0.25 | 0.99 | 0 | 10 | -0.026 | 0.023 | 0.25 | 0.018 | 0.021 | 0.40 | -0.002 | 0.016 | 0.89 |

Decaffeinated tea consumption calculated as the number of cups of decaffeinated tea per day. Time reflects data collected during pregnancy at 8, 18 and 32 weeks gestation and 2, 47, 85, 97 and 145 months after delivery. Beta reflects the number of drinks per day per T allele. Combined Score reflects the number of T alleles summed across SNPs rs2472297 and rs6968865.
